# Department of Public Safety, Philadelphia

**Published:** 1896-05

**Authors:** 


### DEPARTMENT OF PUBLIC SAFETY, PHILADELPHIA.

### CIVIL-SERVICE EXAMINATION QUESTIONS FOR CONSULTING VETERINARIANS, APRIL 2, 1896.

1. What diseases are directly transmissible from animals to men through consumption of meat? 2. What conditions render meat dangerous as food where disease is not directly transmissible? 3. Describe the causes and lesions of actinomycosis. 4. What is "monkey-veal," and in what way is it objectionable? 5. How may the approximate age of a veal-carcass be determined? 6. What are the lesions of hog-cholera? 7. What organs are chiefly affected in tuberculous hogs? 8. How may trichinosis of hogs be recognized? Give the method of examination in detail? 9. Mention the various parts of the body that may be the seat of tubercles in cattle, in the order of the frequency of their infection? 10. How may horse-meat be distinguished from beef?

